# Statistical analysis of comet assay results

**DOI:** 10.3389/fgene.2014.00292

**Published:** 2014-08-27

**Authors:** Peter Møller, Steffen Loft

**Affiliations:** Section of Environmental Health, Department of Public Health, University of CopenhagenCopenhagen, Denmark

**Keywords:** ANOVA, comet assay, experimental design, non-parametric test, statistical analysis

The comet assay can distinguish small differences in DNA damage between different samples of cells, implying that statistical tests are important to assess whether this occurs by chance. Excellent scholarly papers with concise descriptions of statistical analysis and recommendations for tests have been published (Lovell et al., 1999; Lovell and Omori, 2008). We often come across publications that unfortunately have not taken advantage of statistical models in design and analysis of comet assay results. The present commentary is based on the notion that statistical analysis of comet assay data should not be complicated, but consideration of statistical analysis before carrying out the experiments typically makes it much easier to analyse the results.

## Why do we perform statistical analysis of comet assay results?

Statistics are typically done to prove that the DNA damage levels are different between groups, although we formally test for no difference between groups. By default the probability of rejecting the null hypothesis is 5%, although this value is not sacrosanct. Nevertheless, *P*-values less than 5% can make the difference between publishing in prestigious journals or not. Therefore, there is a certain impetus toward producing low *P*-values and misconception of what it really means.

### Misconception 1

The *P*-value (e.g., *P* < 0.05) does not indicate that probability of the null hypothesis being true (i.e., P(H_0_|R) < 5%). On the contrary, the *P*-value is the probability of the observed result given the null hypothesis is true (i.e., P(R|H_0_) < 5%). It means that if we did the experiments again, there would be less than 5% chance that the DNA damage level was the same between groups.

### Misconception 2

The *P*-value does not describe the magnitude of biological effects, because it depends on the variation of DNA damage and number of observations. Datasets with little standard deviation and large number of observations can be highly significant in statistical analysis.

### Misconception 3

The *P*-value does not indicate strength of the association between exposure and DNA damage because it depends on the experimental design. For instance, *P*-values from experimental designs with multiple groups or interactions are much more convincing than simple designs with only two groups. In addition, the *P*-value from parametric tests tends to be more convincing than non-parametric tests.

## What is the experimental unit?

In a traditional comet assay study the investigator measures DNA migration in a number of *Comets* from each *Sample* (e.g., blood sample or tissue from one individual). *Samples* in cell culture experiments refer to independent experiments on different days, preferably with cells from different passage number or donors. It is common practice to measure DNA migration in at least 50 *Comets* per *Gel*. There are often two replicate *Gels* per experiment (i.e., one day of analysis). Consequently, there are usually 100 measurements of DNA migration per *Sample*. This is described as a hierarchical nested experimental design where *Comets* are nested within *Gels*, *Gels* are nested within *Samples*, and *Samples* are nested within *Treatment*. However, it is very important to acknowledge that *Comets* in the same gel have been subjected to the same assay procedure and they are therefore not independent observations. Inclusion of all *Comets* in the statistical analysis is therefore a severe violation of the principle assumption that the statistical analysis is based on independent observations. When evaluating *in vivo* data, the animal is the experimental unit.

The issue about the experimental unit was already discussed extensively in the 1990s and it was clearly stated that “the sample rather than the cell is the experimental unit” (Lovell et al., 1999). Nevertheless, it appears that certain investigators integrate individual *Comets* in the statistical analysis (Bright et al., 2011). Unfortunately, it appears that commercial suppliers also use individual *Comet* data in their instruction for comet assay analysis (e.g., Trevigen Instructions, Catalog #4256-010-CC).

## What is a statistical analysis?

The statistical analysis basically compares the variation between known variables (e.g., exposure groups) with residual variation (e.g., assay variation). However, we rarely know the residual variation and therefore assess it in the same experiment as the known variables. Therefore, it is best to have as many data in the statistical analysis as possible because it provides a better determination of the residual variation. In the statistical analysis, we first calculate the total variation, thereafter the variation related to the known variables, and this subtracted from the total variation should give the residual variation. Because of this procedure, the variation within different groups should be similar (i.e., homogeneity of variance). In addition, the residuals (i.e., difference between the observed and expected value, based on the statistical model) should have a normal distribution because it principally is caused by random variation.

## Can parametric tests be used for comet assay data?

**Figure 1:**
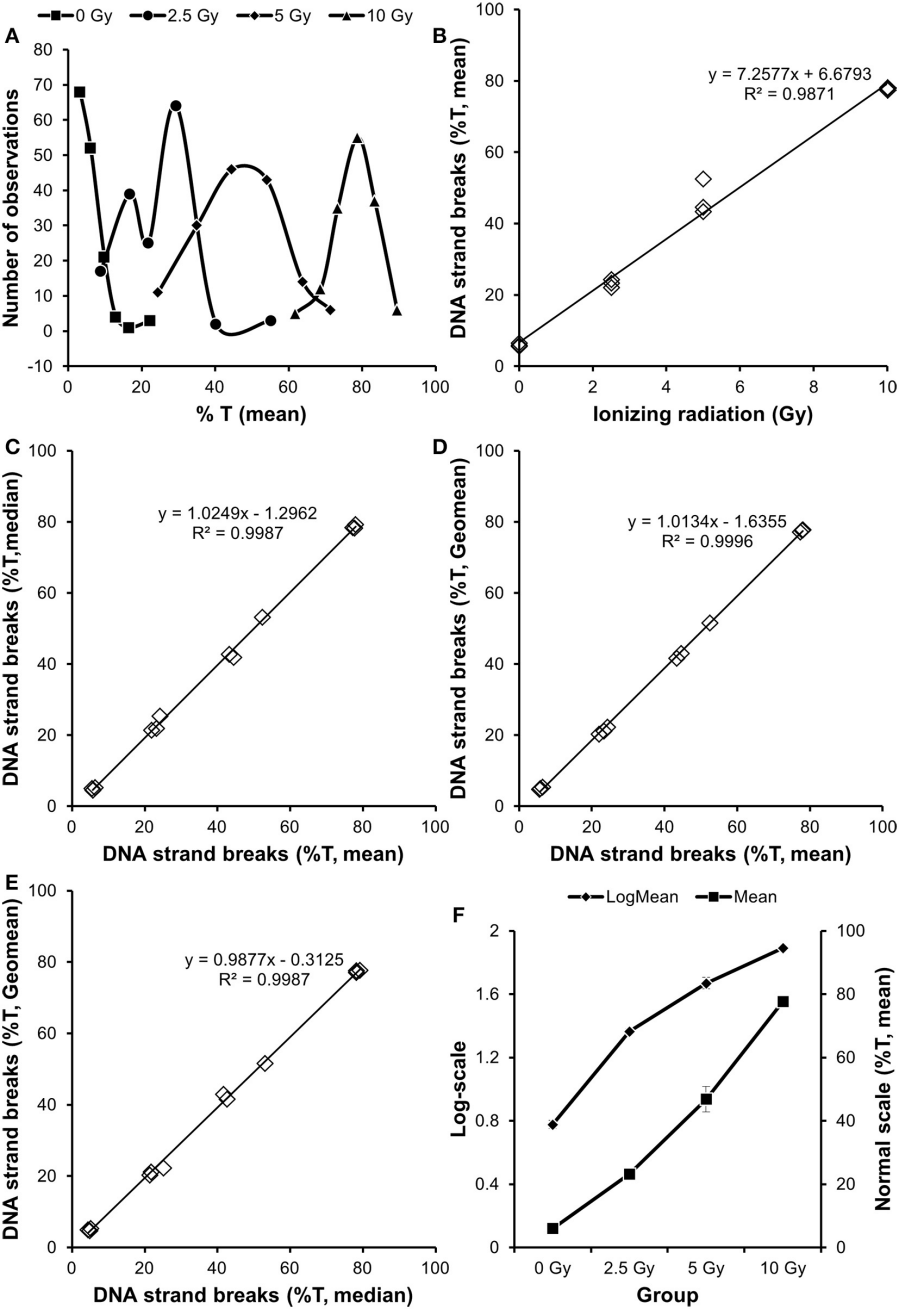
**Analysis of association between exposure to ionizing radiation and level of DNA damage**. The mean values from individual *Comets* displayed variation that tended to shift dose-dependently from non-normal distribution to normal distribution **(A)**. The dataset consisted of 3 independent *Samples* per *Treatment* **(B)**. There was no difference whether the data in *Sample* was obtained from the mean, median of geometric mean of the individual *Comets* **(C–E)**. An analysis of the data by ANOVA indicated inhomogeneity of variance between groups, which was diminished by log-transformation **(F)**.

The distribution of *Comets* is typically non-normal. This sometimes leads to the misconception that comet assay data cannot be analyzed by parametric tests. As an example, Figure 1 outlines a dataset of human peripheral mononuclear blood cells that have been exposed to ionizing radiation. This statistical analysis is applicable to cell culture, animal and human results. There are 3 *Samples* for each ionizing radiation dose, each *Sample* being the data derived from measuring DNA damage in 50 *Comets*. As example of a statistical question, we want to assess the magnitude of effect generated by 5 Gy of ionizing radiation in cellular DNA damage.

Figure 1A reveals that the distribution of *Comets* is non-normal at low doses, while it seems to follow the normal distribution at high doses. Figure 1B shows the dose-response relationship, each symbol being the mean value of the individual *Comets*. Although there are different distributions of individual *Comets*, there is a linear relationship between the radiation dose and DNA damage level. Figures 1C–E display a high correlation between values that have been obtained from the mean, median or geometric mean of the individual *Comets*. Indeed, it makes little difference using the mean or median of *Comets* of even highly skewed distributions in the present dataset.

The data in Figure 1B can be analyzed by either parametric or non-parametric tests, depending on the homogeneity of variance and distribution of residuals (i.e., the unexplained variation). There are a range of different *post-hoc* testsparametric tests, including Dunnett's, Fisher's least statistical difference, Scheffe's and Tukey's tests. Given a hypothesis of a linear relationship between the dose and DNA damage, these data can be analyzed by regression analysis. However, we will in this example use one-way analysis of variance (ANOVA), implying no *a priori* hypothesis of a linear relationship. First we test for homogeneity of variance between the groups (e.g., by Levene's test). In this case, there is inhomogeneity of variance (*P* = 0.005). One result at 5 Gy is aberrant, which is easily demonstrated by substituting it with a dummy variable (i.e., the mean of the two other data points at 5 Gy, *P* = 0.38). Importantly, the aberrant value is higher than expected, which could be a problem because the statistical analysis may show significance due to this value only, while it does not look like an outlier. A log-transformation of the data reduces the inhomogeneity of variance (*P* = 0.044), although principally it still violates the assumption for parametric tests. One option would be to analyse the data with a non-parametric test (Kruskal-Wallis tests of ranks). This shows statistically significant (*P* < 0.0156), but a *post-hoc* Tukey-type comparison test among medians indicate that 0 and 2.5 Gy (as well as 5 and 10 Gy) are not different. Thus, a non-parametric analysis of the data is not an optimal solution and we wanted to assess the magnitude of effect. Therefore, we proceed with a parametric ANOVA, knowing the potential bias due to the aberrant value. The overall ANOVA is highly significant (*P* < 0.001). A *post-hoc* calculation of the fold-difference and 95% confidence interval (CI) shows 7.8-fold (95% CI: 7.0-8.6 fold) increased level of DNA damage at 5 Gy for data assessed on normal scale, whereas a back-transformation of the log-transformed data yields the same mean fold-difference with a slightly larger and skewed CI (6.9–8.9 fold). The CI is also larger when calculated from the standard deviation of only the three 5 Gy results (5.8–9.9 fold), although it is still highly significant as it does not include unity (unit = 1).

Overall, this example demonstrates that one can do a reliable statistical analysis on even non-optimal datasets. However, it should be emphasized that the dataset was balanced (i.e., equal number of observations in each group), whereas this may not hold true for especially datasets with uneven number of observations between groups.

## What type of statistical analysis should be used?

It should be emphasized that having chosen the statistical design before starting the experiments is a huge advantage. The type of design surely depends on the research question, but usually economic issues are important too. For instance, experiments with 4 independent variables would add up to 64 different groups in a simple full factorial design (4^4^-groups). Here we describe three examples for experiments with special emphasis on the research question and study design.

### Example 1: are particles from combustion of biodiesel less genotoxic than conventional diesel?

To answer that question, we investigated DNA damage by particles obtained from combustion of different types of diesel in two different engines, which essentially comply with previous and present EU regulation. In addition, a reference material was included in the experiments and samples were tested in three different concentrations (Hemmingsen et al., 2011). In this design there are numerous irrelevant comparisons (e.g., high concentration of reference material against low concentration of particles from an engine complying with present EU regulation). However, we also wanted to have all data in the same model because it increases the statistical power by better determination of the residual variation. Consequently, these results were tested with nested ANOVA where concentrations were nested in particles.

### Example 2: do dyslipidemic mice have higher age-dependent accumulation of DNA damage than normal mice?

The question entailed a combination of linear (age) and categorical (strain) independent variables. Therefore, it was analyzed with a generalized linear model, assessing the interaction between age and strains. It showed that the two strains of mice had similar accumulation of strand breaks in the liver (single-factor effect of age), whereas there was an interaction between age and strain for oxidatively damaged DNA so that dyslipidemic mice had a higher regression coefficient as compared to wild-type mice (Folkmann et al., 2007).

### Example 3: is exposure to sunlight associated with increased level of DNA damage?

The exposure to sunlight in Denmark is characterized by periods of high exposure (i.e., summer days with sunshine). This exposure was investigated in a repeated measurement study where subjects were followed for 14 months (Møller et al., 2002). Each subject was asked to give blood approximately every third week. However, the data could not be analyzed by repeated measurement ANOVA because of unequal periods of sampling for each subject and it was important to adjust for potential confounders. Therefore, these data were analyzed with a generalized linear mixed model robust to unequal timescales, with demographic variables, nutrition, exercise, and sunlight exposure as independent variables. In addition, the DNA damage levels were assessed on fresh blood samples, together with cryopreserved control samples. The statistical analysis showed that sunlight intensity, hours spent in the sun, and sex were statistically significant variables. The remaining variation (standard deviation of residuals) was the same as the variation in the control samples, indicating that the other variables in the statistical model had no effect on the level of DNA damage.

Collectively, comet assay data can be analyzed by parametric and non-parametric tests. We recommend that the experimental design determines the type of statistical analysis and balanced designs are more robust to datasets with inhomogeneity of variance between groups or non-normal distribution of residuals.

### Conflict of interest statement

The authors declare that the research was conducted in the absence of any commercial or financial relationships that could be construed as a potential conflict of interest.
